# Correlation of three dimensional anorectal manometry and three dimensional endoanal ultrasound findings in primi gravida: a cross sectional study

**DOI:** 10.1186/s13104-015-1346-y

**Published:** 2015-08-29

**Authors:** Dakshitha Praneeth Wickramasinghe, Chamila Sudarshi Perera, Hemantha Senanayake, Dharmabandhu Nandadeva Samarasekera

**Affiliations:** Department of Surgery, Faculty of Medicine, University of Colombo, Kynsey Road, Colombo 8, Sri Lanka; Department of Obstetrics and Gynaecology, Faculty of Medicine, University of Colombo, Kynsey Road, Colombo 8, Sri Lanka

**Keywords:** 3D anorectal manometry, 3D endoanal ultrasound, Correlation, Primi gravida, Anal sphincters

## Abstract

**Background:**

3-dimensional anorectal manometry (3DARM) and 3-dimensional endoanal ultrasound (3DEAUS) have not been used to assess the anal sphincter complex (ASC) in primi gravida. This study was conducted to identify any correlation that may exist between 3DARM and 3DEAUS.

**Methods:**

We analyzed 3DARM and 3DEAUS data of 101 consecutive primi mothers assessed in the late second trimester or early 3rd trimester. 3DARM was performed using the Given Imaging^®^ Manoscan system and 3DEAUS was performed with the Olympus^®^ RU 12M-R1 probe and EU-ME1 ultrasound system.

**Results:**

The mean age was 24.7 (SD—5.1) years. All patients had a normal Cleveland Clinic Incontinence Score. The mean resting pressure (RP) was 87.02 (SD—18.43) mmHg and the maximum squeeze pressure (SP) was 179.21 (SD—52.96) mmHg. The mean length of the high pressure zone was 3.67 (SD—0.52) cm. On 3DEAUS, there were three characteristic segments of the ASC that were identified; upper, middle and lower. Mean thicknesses for both internal anal sphincter (IAS) and external anal sphincter (EAS) were identified for primi gravida. IAS was thicker anteriorly and at 9 o’ clock positions and EAS was thicker posteriorly. There was good correlation in the length of the ASC at each quadrant between 3DARM and 3DEAUS. There was no correlation between either RP or SP thickness of IAS or EAS at each level and quadrant.

**Conclusion:**

Correlation is seen only in the length of ASC at each quadrant. No correlation exist between RP or SP and thickness of IAS and EAS.

## Background

Galen and Versalius (as quoted by Wexner) [1] are acknowledged as the first to describe and illustrate the anal sphincter complex (ASC). First attempts at assessing the anal sphincter pressures were made more than half a century ago [2] and anorectal manometry (ARM) was first used in assessing patients in the 1980s. These devices evolved from intraluminal balloons [3] to water perfused [4] and solid state [5] manometers and finally high resolution anorectal manometry (HRARM) in 2007 [6] and three dimensional (3D) anorectal manometry (3DARM) in 2010 [7]. 3DARM is the only investigation that provides sufficient radial resolution to allow accurate, simultaneous 360° assessment of the ASC. 3DARM being a static test minimizes motion artefacts and other confounders

Endoscopic ultrasound was first used in the assessment of the ASC in 1990 at the St. Mark’s Hospital, London, UK [8]. It showed a good correlation with electromyography (EMG) [9] which was considered the gold standard at the time. EAUS also has a good correlation with surgical findings [10] and histological/dissection studies [11]. Three dimensional EAUS (3DEAUS) was used for patient assessment a decade later [12–14].

The available data on pregnant mothers with either investigation are scant. Authors who have looked at normal values of 3DARM have completely excluded pregnant mothers from their studies or only assessed them postpartum. Authors who have looked at normal values of 3DEUS have not included primigravida or pregnant mothers [15]. In addition, though several authors have compared ARM and EAUS in the assessment of ASC [16] in patients with sphincter damage, 3DARM and 3DEAUS findings of healthy adults have not been compared previously.

The primary objective of this study was to describe the correlations of 3DARM and 3DEAUS in a cohort of healthy primi gravid mothers.

## Methods

### Population and sample

101 consecutive primi mothers who presented to the antenatal clinic of the University Obstetric and Gynaecology unit of the Faculty of Medicine, University of Colombo, Sri Lanka, between May and December 2012 were recruited to the study. They all underwent the assessment between 30 and 32 weeks of gestation. Basic demographic details and details of the gestation were also recorded at the first visit. Anal continence was assessed clinically using the Cleveland Clinic Incontinence Score [17]. Exclusion criteria were patients with symptoms or treatment for any anorectal diseases, previous anorectal trauma or surgery or a previous diagnosis of a neuropathy or anal sphincter dysfunction.

Manoeuvring on the examination bed and lying on the left lateral position for about half an hour would be troublesome in the last part of the pregnancy. An earlier evaluation (1st or 2nd trimester) was possible but would run the risk of loss of follow-up either due to change of residence or Obstetrician, as well as a stillbirth. Therefore we chose to evaluate them between 25th to 32nd weeks of gestation.

The patients were assessed without using any bowel preparation. Both procedures were carried out on the same day with 3DARM being done first in all patients.

Informed written consent was taken from all participants. Ethical approval was obtained from the Ethics Review Committee of the Faculty of Medicine, University of Colombo, Sri Lanka.

### 3DARM equipment and protocol

3D ARM was performed with the patient in the left lateral position. We used the ManoScan AR system by Given Imaging (Yoqneam, Israel). The manometry probe is 10 cm in length and 10.75 mm in diameter. There are 256 transducers in the pressure-sensitive part of probe which is 64 mm in length and arranged in 16 rows with each row having 16 circumferentially arranged sensors. The software linearly interpolate the spaces between the sensors to form a continuous grid. The pressure plots are displayed in the proprietary software (Manoview AR, Given Imaging, Yoqneam, Israel) [13].

The probe was inserted into the anal canal after lubricating and positioned to place the high pressure zone (HPZ) in the middle of the pressure sensitive part with the orientation bump at 6 o’ clock. After allowing the anal canal pressures to stabilize, several parameters were assessed in the following order: resting pressure (RP) (one measurement lasting 20 s) and squeeze pressure (SP) (three attempts for a duration of 20 s each), the recto-anal inhibitory reflex (RAIR) and rectal sensation were simultaneously evaluated by distending the rectal balloon in 10-ml increments up to 200 ml or maximum tolerated volume. Threshold volumes for first sensation, urgency, and maximum discomfort were recorded.

### EAUS study protocol

EAUS was performed using the 360° 10 MHz Olympus GFUM 20 endoanal probe (Olympus America Inc., Pennsylvania). In this probe, the ultrasound sensor mechanically rotates through 360° inside a plastic cylinder. Both these factors (10 vs 7 MHz and sensor inside the plastic cylinder vs water-filled balloon) increase the image quality [18]. The scanning was carried out with the patient in the left lateral position without sedation. Thicknesses of IAS was measured at mid sphincter level at 3, 6, 9 and 12 o’ clock positions and the thicknesses of EAS was measured at upper, middle and lower sphincter levels at 3, 6, 9 and 12 o’ clock positions. Thickness of PRM was measured at 6, 9 and 12 o’ clock positions. After obtaining 2 dimensional (2D) images at the above 3 levels the 3 dimensional (3D) image was acquired. All data were stored on the EU-ME1 receiver.

### Statistical analysis

The data was recorded and analyzed using SPSS version 20 (IBM Corp. Released 2011. IBM SPSS Statistics for Windows, Version 20.0. Armonk, NY: IBM Corp.).

All continuous data are described with the mean and standard deviation. Correlations were identified using Pearson correlation coefficient (Pearson ρ).

## Results

### Participants

There were 101 primi gravida who underwent both 3DEAUS and 3DARM. The mean age was 24.7 (SD 5.1) years. The mean height was 153.9 (SD—5.7) cm and the mean weight at the time of assessment was 59.4 (SD—10.3) kg.

All patients had a normal Cleveland Clinic Incontinence Score.

### 3DARM values and correlations

The mean RP was 87.02 (SD 18.43) mmHg and the maximum SP was 179.21 (SD–52.96) mmHg. The mean length of the HPZ was 3.67 (SD—0.52) cm. Mean volumes for initial rectal sensation, urge and discomfort were 50.36 ± 25.57, 76.70 ± 35.17 and 143.40 ± 66.26 ml respectively.

The pressure asymmetry was highest in the lower anal sphincter where difference between the values as expressed as a proportion of the highest value was approximately 40 % for RP and 50 % for SP. The asymmetry was lowest in the mid sphincter where the same proportions were approximately 4 percent for RP and 10 % for SP.

There was a statistically significant inverse relationship between the length of the high pressure zone (LHPZ) and the mean RP (Pearson ρ −.23, *p* = 0.01). There also were statistically significant correlations between LHPZ and the height (Pearson ρ .22, *p* = 0.028) and weight (Pearson ρ .25, *p* = 0.012).

### 3DEAUS values and correlations

The IAS was seen as a hypoechoic circular tube that continues from the inner circular muscular layer of rectum above and ends in the lower part of mid-anal canal. The EAS had three parts: deep/puborectalis (PRM), superficial and subcutaneous.

The mean thickness of PRM at 3, 6 and 9 o’ clock positions were 7.37 (SD—1.45) mm, 7.21 (SD—1.62) mm and 7.43 (SD—1.40) mm respectively.

At the mid sphincter level (MSL), mean thickness of IAS at 3, 6, 9 and 12 o’ clock positions were 1.77 (SD—0.59) mm, 1.57 (SD—0.57) mm, 1.65 (SD—0.55) mm and 1.72 (SD—0.53) mm respectively. At the same level, the mean thickness of EAS at 3, 6, 9 and 12 o’ clock positions were 5.18 (SD—1.21) mm, 4.77 (SD—3.39) mm, 5.26 (SD—1.37) mm and 3.87 (SD—1.38) mm respectively.

At the lower sphincter level (LSL) EAS thickness at 3, 6, 9 and 12 o’ clock positions were 6.01 (SD—1.06) mm, 6.23 (SD—1.23) mm, 5.94 (SD—1.15) mm and 4.48 (SD—1.12) mm respectively.

There was a statistically significant difference in thickness at 6 and 12 o’ clock positions of the IAS where the IAS was thicker anteriorly (Wilcoxon signed-rank test, *Z* = −2.642, *p* = 0.008). The EAS at both MSL ((Wilcoxon signed-rank test, *Z* = −3.70, *p* < 0.001) and LSL (Wilcoxon signed-rank test, *Z* = −7.712, *p* < 0.001) was thicker posteriorly. The only statistically significant difference in thicknesses between 3 and 9 o’ clock positions was seen in the IAS (Wilcoxon signed-rank test, *Z* = −2.081, *p* = 0.037) where it was thicker at 9 o’ clock position. The EAS (including the PRM) showed good symmetry at all three levels.

The thickness of the IAS at mid sphincter level had a statistically significant correlation with age (Pearson ρ .37, *p* < 0.001).

No length measurements could be obtained in 3DEAUS as the probe was manually withdrawn. However, visual analysis identified that both IAS and EAS were shortest anteriorly.

### Correlations between 3DARM and 3DEAUS

On both 3DARM and 3DEAUS, the ASC was longest laterally (3 and 9 o’ clock positions), followed by posteriorly (6 o’ clock position) and shortest anteriorly (12 o’ clock position).

There were no correlations between the RP and thickness of IAS, and SP and thickness of EAS at the corresponding segment and level.

## Discussion

In patients with symptoms of anorectal disease, assessment of the structure and function of the ASC is an essential step in the evaluation. The American Gastroenterological Association has identified the usefulness of anorectal manometry and endoanal ultrasound in the evaluation of anorectal symptoms and disorders [18]. The normal ranges for 3DARM and 3DEAUS must first be identified to identify abnormalities. The available data in manometry are primarily for high resolution (non-3D) manometry [14, 16] and shows considerable heterogeneity in values, which vary with age, gender, height and ethnicity.

Our results indicate that in Asian primi gravida, the RP, SP and HPZ length are comparable but higher than the values for other Asian females [13] but similar to Caucasian females aged less than 50 years [14]. There was circumferential and axial asymmetry, highest in the LSL and lowest in the MSL, mean thicknesses of IAS, EAS at all levels and PRM are less than other Asian females [15]. There was also no asymmetry of the EAS at 3 and 9 o’ clock positions but there is lateral asymmetry in the IAS where it is thicker at 9 o’ clock position, The ASC was longest laterally as identified by both 3DARM and 3DEAUS, and there was no correlation with RP and SP with thickness of either sphincter at the corresponding level.

Our selection of antenatal participants serves several purposes. In a practical setting, females who are not at increased risk of anal incontinence neither present for anorectal physiology testing before conception, nor is it indicated unless there is overwhelming suspicion of an anal sphincter injury. Therefore, we wanted to describe the parameters of the ASC in primi gravida at a time frame comparable to their usual presentation. Furthermore, to our knowledge, this is the first time 3D ARM and 3DEAUS have been concurrently performed on primi gravida during the antenatal period. Because both these investigations are more sensitive, occult changes could’ve been identified. Although early investigators concluded that pregnancy itself does not influence the morphology or function of the ASC [19], anal incontinence has been described even in mothers who underwent elective caesarean deliveries [20, 21]. Therefore, a vaginal delivery is not a prerequisite for obstetric anal sphincter injuries and physiological changes and occult injuries during pregnancy could contribute to these changes.

A recent article has also compared 3DARM with 3D ultrasound, but using trans-perineal ultrasound [22]. EAUS is considered the gold standard in evaluating anatomical abnormalities of the anal sphincter in the evaluation of incontinence [23]. The problems of using trans-perineal ultrasound include poor visualisation of the lateral border of the EAS, and the inability to visualize anal mucosa and submucosa separately [24]. Trans-perineal ultrasound is also less sensitive in detecting sphincter injuries [25]. Furthermore, the authors do not provide values for thickness of IAS or EAS, which are the common parameters used in patient assessment. To our knowledge, this article is the first to compare 3DARM and 3DEAUS, both being the gold standard in their own arena.

In addition to the quantitative changes seen after delivery, Titi et al. [26] describes qualitative changes in both IAS and EAS. They assessed the uniformity of echogenicity of the sphincters and classified them as good quality (uniform echogenicity) or poor quality (mixed echogenicity) and found that the quality of EAS did not affect the SP but the quality of IAS affected the RP. One possibility for this disparity is the characteristic appearance of skeletal and smooth muscle as well as fibrous tissue on ultrasound. Injury to permanent cells heal by fibrosis. Fibrosis appears hyperechoic on ultrasound [27]. EAS, which is comprised of skeletal muscle also appears hyperechoic on ultrasound whereas predominantly smooth muscle containing IAS appears hypoechoeic on ultrasound [28]. Therefore, there will be better delineation of fibrosis of IAS than fibrosis of EAS on EAUS.

We did not attempt to assess the quality of the sphincter echogenicity on EAUS for several reasons. The above results have not been replicated by subsequent studies and there is no universally accepted criteria on the assessment of echogenicity. Furthermore, there are no therapeutic options to improve the overall quality of the muscle [26] even if a change is identified. Therefore the assessment offers no therapeutic advantage at present.

The participants of our study were all Sri Lankan primi gravida. Nevertheless, this is the first study to describe 3DARM and 3DEAUS in pregnant women or nullipara. All previous studies have excluded this category [13–16, 22]. Childbirth is a well-established risk factor for ASC injuries, especially in primi gravida [29]. Occult obstetric anal sphincter injuries detected on EAUS may be as high as 67–92 % [30]. Hence, our data would be useful in assessing primi gravida for both research and clinical purposes.

Previous authors have found that every ASC defect seen in 3DARM was also seen in 3DEAUS [16] but the authors state that the level of agreement between the 2 was inadequate in the diagnosis of anal sphincter defect for 3DARM to be used routinely in the diagnosis. Our findings confirm that this conclusion is valid in the assessment of patients with undamaged anal sphincters (e.g.—primi gravida) as well. This is also in keeping with the findings of previous studies utilizing non-high resolution manometry and 2D endoanal ultrasound [26, 31].

The absence of correlation between pressures measured with 3DARM and sphincter thickness measured by 3DEAUS is surprising, especially because the asymmetry of ASC was seen in both and the length of ASC in different quadrants also correlated well. Findings of previous authors using non-3D methods have also been divided, with data showing both good agreement when assessing patients with anorectal disease [32] and no correlation when assessing normal patients [31]. It is possible that given the high sensitivity of both these investigations, they identify all structural and functional defects in the ASC and therefore has a good correlation in assessing patients with damage to ASC, whereas in normal adults there is poor correlation between the sphincter pressures and sphincter thickness. Other possible reasons are the subjective nature of the EAUS interpretation, operator dependency, normal variations [26] and inability to distinguish muscle fibers from surrounding fibro-fatty tissue [33]. However 3DEAUS is capable of reducing operator dependency [34].

We found that the presence of occult sphincter injuries did not affect the incontinence score. This is similar to the findings of Faridi et al. [35] and Wasserberg et al. [36]. Therefore sphincter lesions after vaginal delivery cannot be excluded by the absence of bowel symptoms postnatally. One reason for this lack of correlation between the presence of occult injuries and absence of symptoms in our study is the age of our population. Of the above compared studies, ours had the lowest mean age. Younger patients are believed to have higher sphincter reserves and therefore better compensation of sphincter injuries. An EAS defect can be compensated with contractions of Puborectalis and pelvic floor muscles [37].

Both 3DARM and 3DEAUS parameters in our study resemble Western values than Asian. One probable explanation is that Sri Lankans (and South Asians) are Caucasoid whereas citizens of the countries where previous articles originated [13, 15] are mongoloid [38].

We were not able to describe the 3DARM and 3DEAUS in a normal cohort, which would’ve enabled a comparison with primigravida. We are presently recruiting participants to establish a set of normal values which will enable us to further strengthen our observations.

## Conclusions

Both 3DARM and 3DEAUS values in Sri Lankan primigravida correlate better with values reported in the West than in Asia. The normal pressure morphology described by previous authors is also well visualized in primigravida. Only the lengths of ASC correlated well between 3DARM and 3DEAUS. No correlations were identified between pressures and sphincter thicknesses at each level and quadrant.
